# Supplementary material for: Resilience and Self-Esteem Mediated Associations between Childhood Emotional Maltreatment and Aggression in Chinese College Students
Source: Behav Sci (Basel). 2022 Oct 7;12(10):383. doi: 10.3390/bs12100383 (PMC9599015; doi:10.3390/bs12100383)
Supplement: Supplementary file 1 [file behavsci-12-00383-s001.zip › behavsci-1858696-supplementary.pdf]

Supplementary file S1: Emotional Maltreatment in Childhood Trauma Questionnaire-short form

1、When I was growing up, someone in my family called me things like “stupid” or “lazy” or “ugly.”	1	2	3	4	5
2、When I was growing up, there was someone in my family who helped me feel that I was important or special.	1	2	3	4	5
3.When I was growing up, people in my family looked out for each other.	1	2	3	4	5
4、When I was growing up, I thought that my parents wished I had never been born.	1	2	3	4	5
5.When I was growing up, someone in my family loved me.	1	2	3	4	5
6、When I was growing up, people in my family said hurtful or insulting things to me.	1	2	3	4	5
7、When I was growing up, I felt that someone in my family hated me.	1	2	3	4	5
8、When I was growing up, people in my family felt close to each other.	1	2	3	4	5
9、When I was growing up, I believe that I was emotionally abused	1	2	3	4	5
10、When I was growing up, my family was a source of strength and support.	1	2	3	4	5
**Supplementary file S2: The Aggression Questionnaire**					
1、I will not tell to others when I feel blue	1	2	3	4	5
2、I have a lot of mood swings and ups and downs	1	2	3	4	5
3、Sometimes I will lose control to beat others	1	2	3	4	5
4、If I disagree with my friends’ opinion, I will give feedback immediately	1	2	3	4	5
5、My anger comes fast and go fast	1	2	3	4	5
6、I have high levels of jealous	1	2	3	4	5
7、If someone bother me on purpose, I will beat him or her	1	2	3	4	5
8、I like to deny others’ opinion	1	2	3	4	5
9、I feel upset when the things are not go well	1	2	3	4	5
10、I feel I meet much more unfired things	1	2	3	4	5
11、When I angry I will hurt myself due to careless	1	2	3	4	5
12、If someone beat me, I will beat back	1	2	3	4	5
13、When others disturb me, I will blame them without hesitation	1	2	3	4	5
14、I feel like a bomb when I angry, and will boom anytime	1	2	3	4	5
15、I will suspect others’ purposes when strangers treat me too kindly	1	2	3	4	5
16、I fight more than others	1	2	3	4	5
17、I will lost my control to debate when others disagree with me	1	2	3	4	5
18、I have difficulties to control my temper	1	2	3	4	5
19、I will take something to heart	1	2	3	4	5
20、I will use violent to protect my rights when necessary	1	2	3	4	5
21、I easily quarrel with others	1	2	3	4	5
22、I easily breast anger when I see things less than satisfactory	1	2	3	4	5
23、I think someone speak ill of me	1	2	3	4	5
24、If someone bother me at a proper extent, I will fight with others	1	2	3	4	5
25、I will lost my temper without any reason	1	2	3	4	5
26、I think others may have purposes when they treat me too kindly	1	2	3	4	5

27、I will throw out things when I feel very anger	1	2	3	4	5
28、I suspect someone laugh at me	1	2	3	4	5

**Supplementary file S3: Self-esteem Scale**					
1、I think I am a valuable person, at least with the same level to others	1	2	3	4	
2、I think I have many good qualities	1	2	3	4	
3、I think I am a loser ultimately	1	2	3	4	
4、I think I can finish things like most person	1	2	3	4	
5、I think I have few qualities which makes me proud.	1	2	3	4	
6、I have positive feeling to myself	1	2	3	4	
7、I satisfy to myself	1	2	3	4	
8、I hope I can receive more respect	1	2	3	4	
9、I feel worthless sometime	1	2	3	44	
10、I always feel worthless	1	2	3	4	

Supplementary file S4: The Chinese Adolescents Resilience Scale

题目(Subjects)	选项(Options)				
1、I am discouraged by failures	1	2	3	4	5
2、I have difficulty to control my unpleasant emotions	1	2	3	4	5
3、I have specific goals for my life	1	2	3	4	5
4、I will become more mature and experiences when I suffered some frustrations	1	2	3	4	5
5、Failures and frustrations will make me suspect my ability	1	2	3	4	5
6、I cannot find a person all the time when I have met unpleasant things	1	2	3	4	5
7、I have a peer friend who I can share my difficulties	1	2	3	4	5
8、My parents respect my opinion	1	2	3	4	5
9、I do not know who can help me when I have difficulties	1	2	3	4	5
10、I think compare to results, the process may help people grow fast	1	2	3	4	5
11、I will make a plan and a solution when I meet difficulties	1	2	3	4	5
12、I always put things in my heart other than tell to others	1	2	3	4	5
13、I think adversity can inspire people	1	2	3	4	5
14、Adversity may be helpful for person's grow up	1	2	3	4	5
15、My parents always interrupt my opinion	1	2	3	4	5
16、Nobody could listen to me in my family	1	2	3	4	5
17、My parents could not support me at confidences and spirits	1	2	3	4	5
18、I will tell to others when I have problems	1	2	3	4	5
19、My parents never berate me	1	2	3	4	5
20、I will concentrate all my strength when I face difficulties	1	2	3	4	5
21、I need more time to forget the unpleasant things	1	2	3	4	5
22、My parents always encourage me to do my best	1	2	3	4	5
23、I can regulate my emotions in a short time	1	2	3	4	5
24、I will set goals to push me	1	2	3	4	5
25、I think everything has a positive aspect	1	2	3	4	5
